# 98 Where Are the Nurses Going?

**DOI:** 10.1093/jbcr/iraf019.098

**Published:** 2025-04-01

**Authors:** Tiffany Hockenberry, Claudia Islas, Stacey Richerbach, Karen Richey, Kevin Foster

**Affiliations:** Diane & Bruce Halle Arizona Burn Center; Diane & Bruce Halle Arizona Burn Center; Diane & Bruce Halle Arizona Burn Center; Diane & Bruce Halle Arizona Burn Center; Diane & Bruce Halle Arizona Burn Center

## Abstract

**Introduction:**

Nursing plays a crucial role in society and healthcare; and is regarded as the most trusted profession in the world year after year. Despite this, an increasing number of nurses are choosing to leave the bedside or step away from the field entirely. Reduced nursing staff poses significant obstacles to organizations, reducing the ability to provide consistent quality care. Institutions risk instability due to the substantial financial strain, loss of institutional memory, increased workload for remaining staff and lower morale. These challenges pose greater obstacles within a specialty field. The purpose of this study was to evaluate nursing turnover rates with a specific focus on burn nursing.

**Methods:**

This was a retrospective review of RN turnover at our hospital and burn center from 2016 to 2023; including Burn Med-Surg (BMS) and Burn ICU (BI). National turnover data for RN staffing and burn-specific turnover (BST) were used as benchmarks. Actual RN staffing data were used to calculate weighted turnover rates. Descriptive statistics were calculated for turnover rates and yearly changes. One-way ANOVA, followed by a Tukey’s HSD post-hoc analysis was done to identify significant pairwise differences. Paired t-tests were conducted to compare turnover rates between burn and hospital turnover rate.

**Results:**

The average turnover rates for the 8-year period were: overall hospital turnover (23%), BMS (38%), and BI (31%). Year-on-year changes showed variability in burn department turnover. There were no significant differences in turnover rates across departments (p=.168). Paired t-tests indicated that both BMS (p=.075) and BI (p=.081) did not differ significantly from hospital rates. However, weighted turnover rates revealed significant differences between departments (p<.001) with Tukey’s HSD post-hoc confirming that BMS and BI turnover was significantly higher than hospital turnover (p <.001). National BST benchmark comparison showed our burn rates were consistently higher, with increases of 200-240% in two years (2019, 2020). Variance analysis revealed turnover in our burn center had significantly higher variability (variance=.0176) compared to the national BST (variance =.0024).

**Conclusions:**

Local RN turnover from 2016 to 2023 revealed significant variability across burn units with rates consistently exceeding national benchmarks for both general RN and BST. BMS and BI had significantly higher rates than the overall hospital rate. Targeted interventions over the past 8 years to retain burn nurses have been unsuccessful. There is an immediate need for alternative interventions to retain RNs in burn practice, both locally and nationally.

**Applicability of Research to Practice:**

Collaboration between centers to explore solutions to the existing crisis with turnover is urgently needed to sustain quality patient care and employee well-being.

**Funding for the Study:**

N/A

